# Spontaneous pancreatic pseudocyst–portal vein fistula: a rare and potentially life threatening complication of pancreatitis

**DOI:** 10.1308/003588413X13511609955616

**Published:** 2013-01

**Authors:** SS Raza, A Hakeem, M Sheridan, N Ahmad

**Affiliations:** Leeds Teaching Hospitals NHS Trust,UK

**Keywords:** Acute pancreatitis, Pseudocyst, Portal vein, Fistula, Endoscopic retrograde cholangiopancreatography, Puestow procedure

## Abstract

Pseudocyst formation following acute and chronic pancreatitis is a well known complication. A pancreatic pseudocyst fistulating into the portal vein is a rare and potentially fatal complication. We report a case of pancreatic pseudocyst – portal vein fistula, which was managed with a conservative approach.

## Case history

A 45-year-old man presented with severe acute alcoholic pancreatitis. Computed tomography revealed extensive inflammation around the head of the pancreas with a small 1.5cm × 2.3cm area of pancreatic necrosis. The gallbladder, liver and biliary tree were normal. He improved on conservative management and was discharged home with advice to abstain from alcohol.

**Figure 1:**
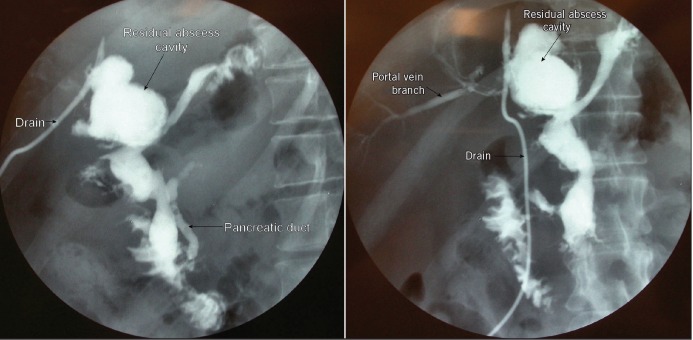
Tubogram through the drain in the left hepatic lobe abscess demonstrating contrast delineating branches of portal vein, second part of duodenum and the pancreatic duct

**Figure 2:**
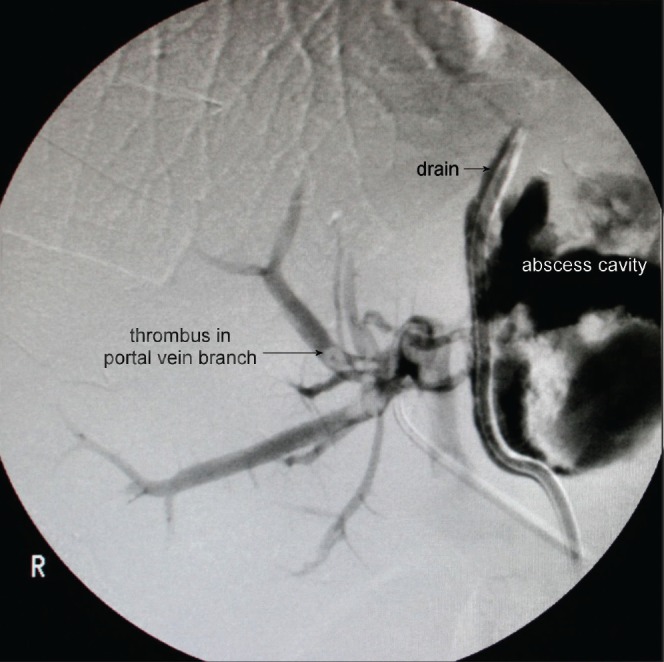
Angiography demonstrating the drain tube abutting the branches of the portal vein and a thrombus in the portal vein branch

The patient presented again two months later with acute pancreatitis (severe on Ranson scoring) and ascites. He underwent ultrasonography-guided drainage of amylase rich ascites and a lesser sac collection. The hospital stay was complicated by acute inferolateral myocardial infarction, which was managed medically. He subsequently required percutaneous drainage of fluid collections and a left lobe liver abscess. A tubogram through the liver drain showed the residual liver abscess cavity communicating with the first part of duodenum with concomitant pancreatography and portal venography (Fig 1). At this stage, he was only draining a small amount of haemoserous fluid. Conventional angiography through the superior mesenteric artery (portal venous phase) showed the drain to be abutting and possibly compromising the branches of the portal vein with a partial occluding thrombus in the left portal vein (Fig 2). A conservative ‘watch-and-manage’ approach was therefore adopted.

**Figure 3:**
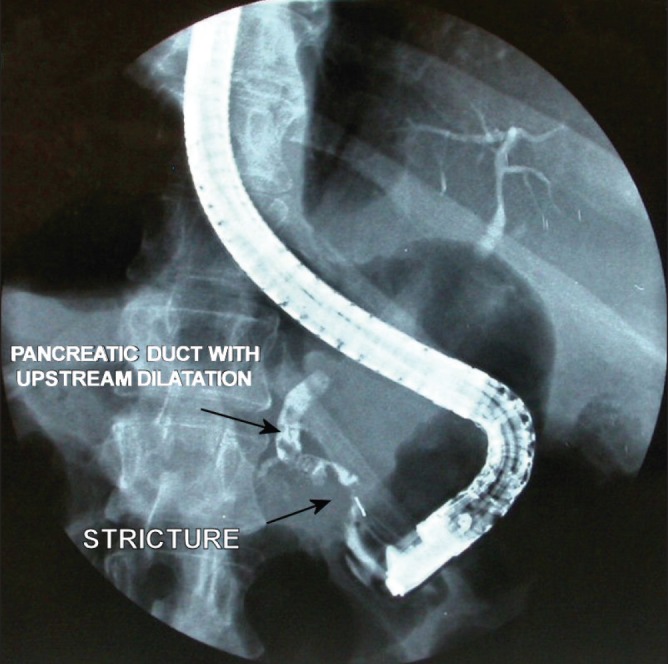
Endoscopic retrograde cholangiopancreatography demonstrating pancreatic duct stricture and multiple filling defects (possibly stones)

Upper gastrointestinal endoscopy (for haemoptysis) showed a large anterior duodenal ulcer with visible blood vessels and active bleeding at the base, which was treated via sclerotherapy. The patient had two further episodes of haematemesis requiring endoscopic management. Once he had stabilised, endoscopic retrograde cholangiopancreatography (ERCP) demonstrated an irregularly dilated duct in the body and tail of the pancreas with multiple filling defects and a possible small segment stricture in the head of the pancreas (Fig 3). The accessory pancreatic duct could not be visualised. A 7cm, 5Fr pigtailed plastic pancreatic stent was inserted in the main pancreatic duct to establish pancreatic drainage. The liver drain was removed and the patient was discharged from hospital three months after his admission with plans for surgery following sufficient recovery from his acute coronary event. He underwent an elective change of the pancreatic stent eight weeks following discharge.

Approximately six months following his initial admission, the patient underwent an elective pancreaticojejunostomy (Puestow procedure) to manage pancreatic duct stricture and establish pancreatic drainage. The surgery was performed through a rooftop incision. A 5cm Roux-en-Y pancreaticojejunostomy to the distal pancreatic duct was performed with interrupted 4/0 PDS® (Ethicon, Somerville, NJ, US) suture and a second layer of continuous 4/0 Vicryl® (Ethicon). The proximal pancreatic duct was strictured and the stent could not be removed. A splenectomy was also performed owing to splenic vein thrombosis.

The patient made a slow but uncomplicated recovery and was discharged home after two weeks. He required another ERCP for stent removal. He then continued to improve and was discharged from follow-up after six months.

## Discussion

Pancreatic pseudocyst–portal vein fistula are a rare but life-threatening sequel of pancreatitis and have been reported previously in the literature.^1–7^ This rare complication seems to effect predominantly men with a strong history of alcohol abuse.^8^

The mechanism by which the pseudocyst fistulates into the portal vein remains unclear. While some postulate thrombosis of the portal vein as the initiating factor, the case mentioned by Dawson *et al* demonstrates portal vein thrombosis developing after the fistula formation.^8^ Although portal vein thrombosis was also demonstrated in our case, the proximity of the drain to the portal vein branches may have contributed to fistula formation by direct erosion.

Most commonly, patients present with acute pancreatitis on a background of chronic pancreatitis but it may be their first presentation with acute pancreatitis. All patients present with abdominal pain with additional signs and symptoms of associated complications such as dyspnoea secondary to pleural fistula or abdominal distension due to ascites. One would expect severe haemorrhage as a result of the fistula with the portal vein but it is interesting that none of the reported cases have described catastrophic haemorrhage secondary to the fistula. One explanation for this may be that the pseudocyst acts as a high pressure system, emptying its contents into the portal vein rather than vice versa.

In the majority of cases, the diagnosis is made by invasive methods such as ERCP or surgery. Four cases report postmortem examination. In only one case diagnosis was made by a non-invasive method such as magnetic resonance cholangiopancreatography. In our patient, the diagnosis was made by a tubogram through a drain in the left liver lobe abscess cavity.

Most reported cases who survived required aggressive surgical treatment including portal vein plasty (for primary closure of the fistula) or pancreatectomy and pancreaticojejunostomy (for blocking the flow of pancreatic juice) as a definitive treatment. In one reported case, a pancreatic stent alone was used to treat the fistula successfully.^7^

Improvement from conservative management is seen only in a minority of patients who are asymptomatic or have mild symptoms. In our case, a catastrophic haemorrhage from the fistula might have been averted owing to the plastic drain abutting the fistula site. The presence of a thrombus in the adjacent portal vein supports this. The stricture of the proximal pancreatic duct may have contributed to the persistence of the pseudocyst and the formation of the pseudocyst–portal vein fistula. ERCP and pancreatic stent insertion were used to effectively block the flow in the pseudocyst and to improve pancreatic drainage. The presence of pancreatic duct stricture with upstream dilatation and stones in the distal pancreatic duct meant that long-term management with a pancreatic stent was not an option. An interval definitive surgical procedure (Puestow procedure) was therefore performed once the patient had recovered from acute events.

## Conclusions

Formation of a pancreatic pseudocyst–portal vein fistula is a rare but life-threatening complication of pancreatitis. A conservative or minimally invasive approach should be adopted initially as surgery carries a high risk of mortality and morbidity. Catastrophic bleeding should be managed radiologically whenever possible with surgery as a last resort. A surgical approach to establish pancreatic duct drainage should be attempted once the acute insult has subsided.
